# Detection of miR-33 Expression and the Verification of Its Target Genes in the Fatty Liver of Geese

**DOI:** 10.3390/ijms160612737

**Published:** 2015-06-05

**Authors:** Yun Zheng, Shibei Jiang, Yihui Zhang, Rui Zhang, Daoqing Gong

**Affiliations:** College of Animal Science and Technology, Yangzhou University, Yangzhou 225009, China; E-Mails: zhengyun@yzu.edu.cn (Yu.Z.); jsb890106@163.com (S.J.); first22686@126.com (Yi.Z.); zrjindy@hotmail.com (R.Z.)

**Keywords:** miR-33, goose fatty liver, target gene, fat metabolism

## Abstract

Background: miRNAs are single-stranded, small RNA molecules with a length of 18–25 nucleotides. They bind to the 3′ untranslated regions of mRNA transcripts to reduce the translation of these transcripts or to cause their degradation. The roles of these molecules differ in biological processes, such as cell differentiation, proliferation, apoptosis and tumor genesis. miRNA-33 is encoded by the gene introns of proteins that bind sterol-regulatory elements. This molecule cooperates with these proteins to control cholesterol homeostasis, fatty acid levels and the genes that are related to the expression of fat metabolism. The examination of miR-33 expression and its target genes can promote the in-depth study of the miRNA regulation mechanism in the formation process of goose fatty liver and can lay a foundation for research into human fatty liver. Methodology/principal findings: (1) Through real-time fluorescent quantitative polymerase chain reaction (TaqMan MicroRNA Assay), we detected the expression of miR-33 during the feeding of Landes geese. The expression level of miR-33 increases significantly in the liver after 19 days in comparison with the control group; (2) By using the bioinformatics software programs TargetScan, miRDB and miRCosm to predict the target genes of miR-33 according to laboratory prophase transcriptome results and references, we screen nine target genes: adenosine triphosphate binding cassette transporters A1, adenosine triphosphate binding cassette transporters G1, Neimann Pick C, carnitine *O*-octanoyltransferase (*CROT*), cyl-CoA dehydrogenase/3-ketoacyl-CoA thiolase/enoyl-CoA hydratase, beta subunit (*HADHB*), AMP-activated protein kinase, alpha subunit 1 (*AMPKα1*), insulin receptor substrate 2, glutamic pyruvate transaminase and adipose differentiation-related protein. The dual luciferase reporter gene system in the CHO cell line verifies that *CROT*, HADHB and *NPC1* are the target genes of miR-33 in geese. The inhibition rate of *CROT* is highest and reaches 70%; (3) The seed sequence (5′ 2–8 bases) is the acting site of miR-33. The two predicted target sites of *CROT* are the target sites of miR-33. Moreover, the predicted target site of *HADHB* and *NPC1* is the target site of miR-33. Conclusions/significance: (1) After 19 days of overfeeding, the expression level of miR-33 increases significantly in the livers of geese; (2) *CROT*, *HADHB* and *NPC1* are the target genes of miR-33 in geese. These genes determine the combined target site.

## 1. Introduction

miRNA is an important control factor in gene expression. Its roles differ in biological processes, such as cell differentiation, proliferation, apoptosis and tumor genesis [1]. Mature miRNA regulates the expression of target genes through complementary pairing with the target mRNA in the target sequence of 3′ untranslated regions (UTR) [2]. In the gene intron of the sterol-regulatory element binding protein (*SREBP*) in fruit flies, mice, chickens, humans and other species, the highly conserved miRNA family miR-33 cooperates with these proteins to form a negative feedback loop. This collaboration also controls cholesterol homeostasis, fatty acid level and the expression of fat metabolism-related genes [3,4,5,6]. Previous studies indicate that the overexpression of miR-33 can reduce the oxidation of fatty acid in liver cells, whereas the inhibition of endogenous miR-33 can increase the expression of carnitine *O*-octanoyltransferase (*CROT*), *CPT1A*, cyl-CoA dehydrogenase/3-ketoacyl-CoA thiolase/enoyl-CoA hydratase, beta subunit (*HADHB*) and AMP-activated protein kinase (*AMPK*). This increased expression enhances fatty acid oxidation [7,8]. Previous research also suggests that the miR-33 can regulate all aspects of fat metabolism by limiting the flow of cholesterol and of fatty acid degradation. miR-33 in the macrophage of mice can be targeted to adenosine triphosphate binding cassette transporters G1 (*ABCG1*) [9] and can promote excessive cholesterol output. However, these transporters act strongly as high-density lipoprotein (HDL) receptors [10,11]. Adenosine triphosphate binding cassette transporters A1 (ABCA1) are a type of membrane-binding protein and a type of cholesterol transporter that can cause excess cholesterol to become extracellular. This process is important in the cholesterol homeostasis of the cells of the entire body. The overexpression of miR-33 can reduce the *ABCA1* mRNA expression in livers and reduce the HDL levels in plasma by 25% [12].

The livers of animals are important to the process of lipid metabolism as the main areas of fatty acid synthesis. In particular, goose liver has a strong capability to deposit fat and can recover after the onset of fatty liver disease. This liver is also immune to cirrhosis and necrosis [13]. From the perspective of medical science, goose liver can be considered a good model for research into the fatty livers of humans and animals. At present, preliminary understanding regarding the molecular mechanism of the onset of fatty liver in geese has been generated [14]; however, the subsequent regulation mechanism remains unclear. Thus, the study on the regulation of miR-33 expression in geese is significant to livestock production and to the treatment of fatty liver disease in humans.

## 2. Results

### 2.1. Precursor Sequence of the miRNA-33 of Landes Geese

**Figure 1:**
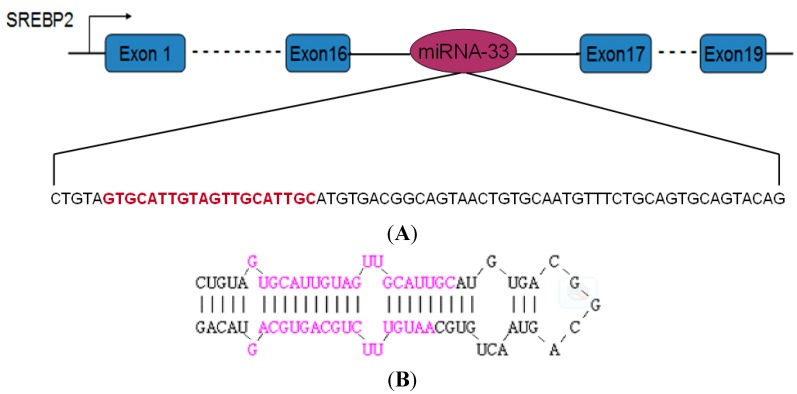
miR-33 precursor sequence and stem ring structure. (**A**) miRNA-33 located in SREBP2 intron 16. The sequence above is the precursor sequence of miRNA-33 in Landes geese. The part of the sequence that is marked in red indicates mature sequences; (**B**) Structure of the stem ring of the miRNA in Landes geese. The part of the sequence that is marked in red represents mature sequences.

The miRNA-33 precursor sequence of geese was amplified using primers designed based on the miRNA-33 precursor sequence of chicken. As per the sequencing results (Figure 1), this sequence included 69 nucleotides. The homology analysis suggested that the maximum homology of geese miRNA reaches 95.65%, unlike that of chicken miRNA. Moreover, geese miRNA contained the complete mature miRNA-33 sequence, which is identical to that of chicken miRNA.

### 2.2. Expression Rule of miR-33 in Goose Fatty Liver

**Figure 2:**
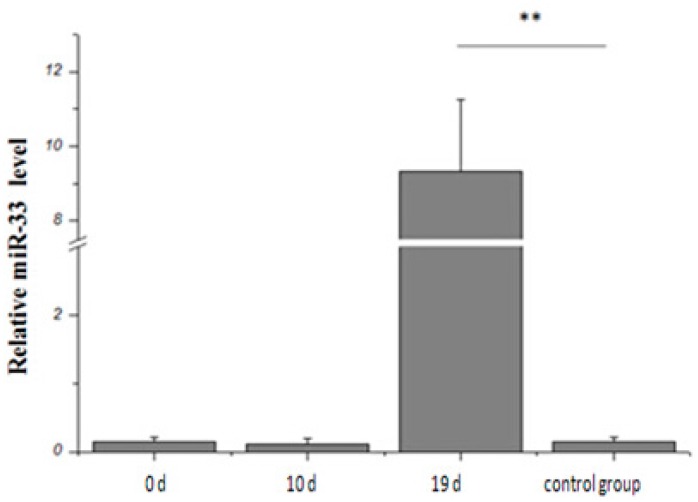
miR-33 expression during the overfeeding of Landes geese. ** represents *p* < 0.01.

The expression of miR-33 in goose liver does not increase after 0 and 10 days of overfeeding (Figure 2). However, this expression increases significantly after 19 days of overfeeding in comparison with the control group.

### 2.3. Prediction of miR-33 Target Genes

**Figure 3:**
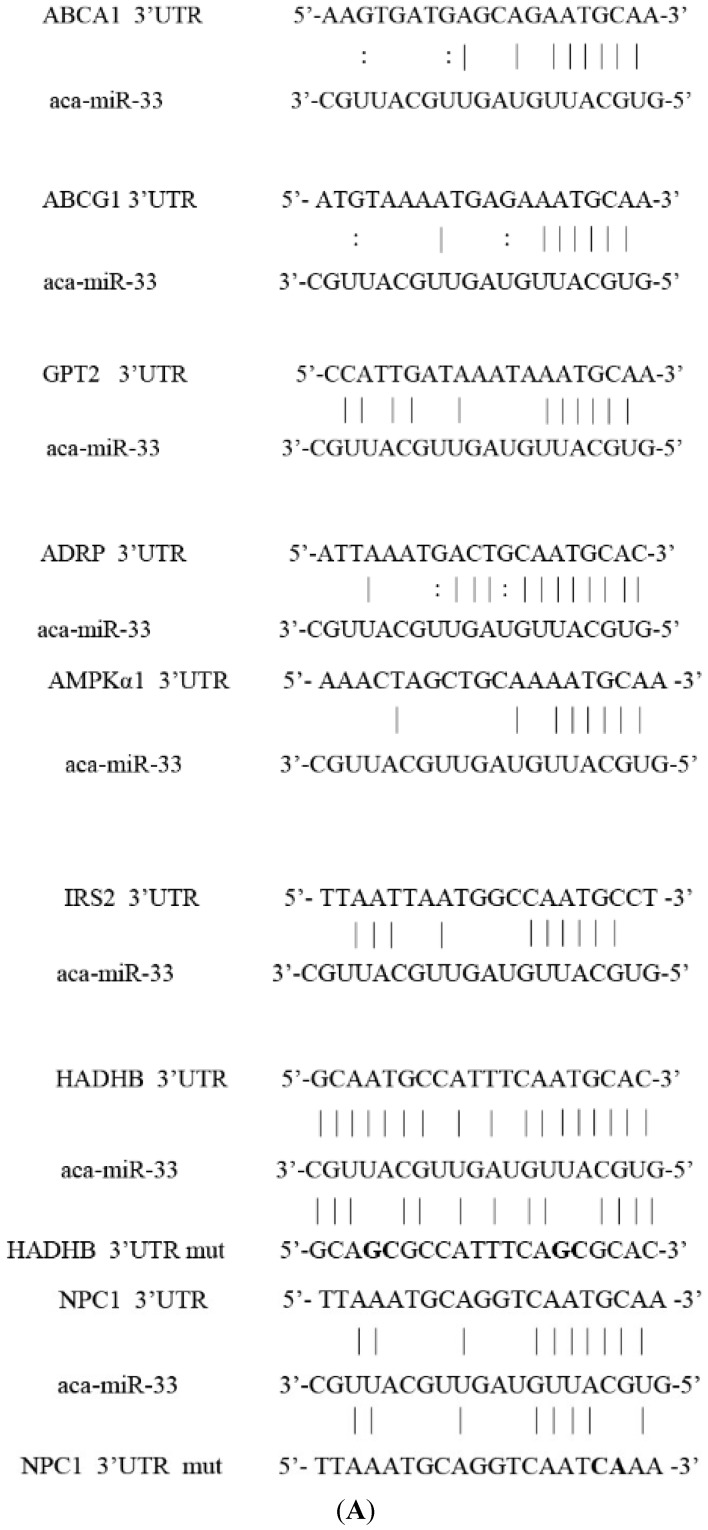

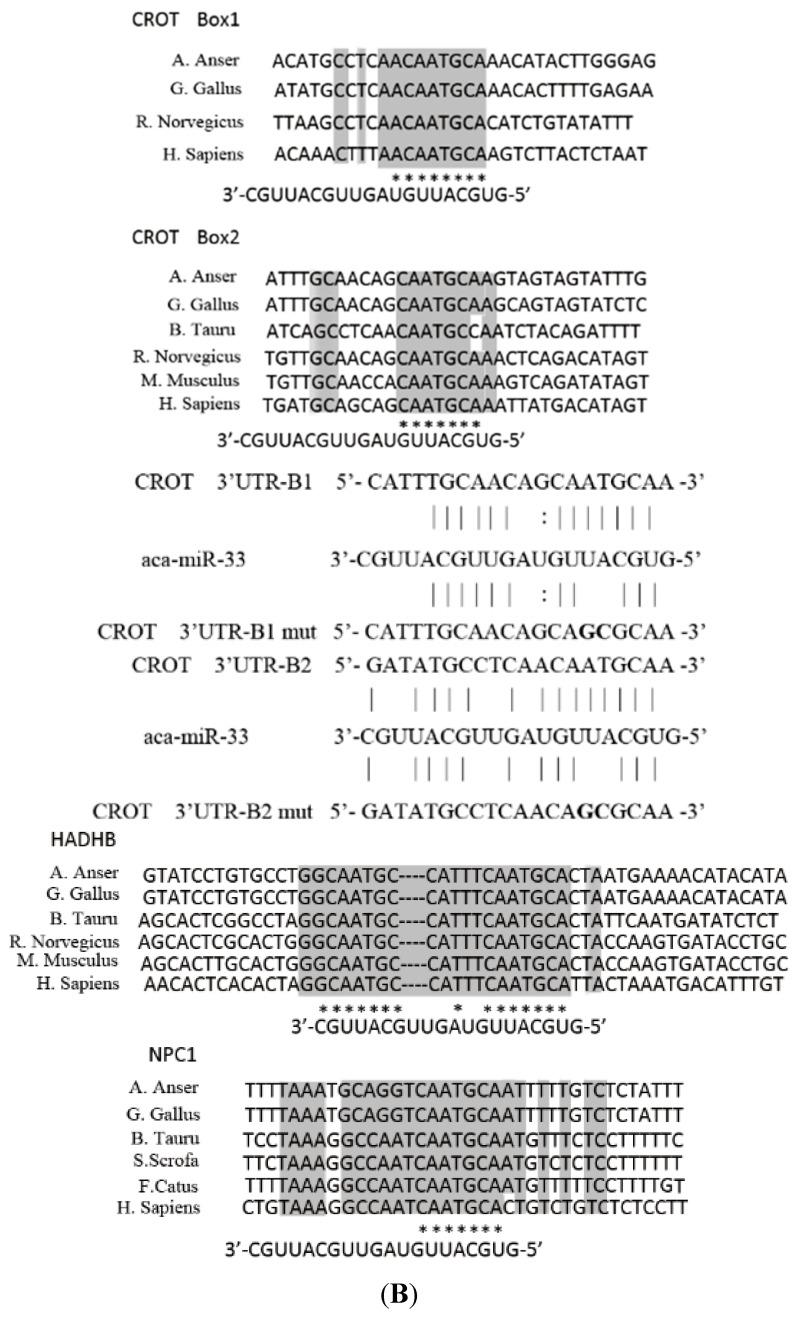
Target binding sites of the miR-33 target gene. (**A**) Complementary situation of miRNA-33 and the target gene loci. Vertical lines represent the complete complement of the genes to one another; “:” represents the GU complement; and the bolded section indicates the bases for mutation. In *HADHB*, the site that is near the 3′ end is target Site 1, and the site near the 5′ end is target Site 2; “aca” represents the geese; “mut” represents the mutation site of target genes; (**B**) Homology in different species of target gene loci. The sequence of the gray area is constant. * The complement base. (*H. Sapiens*, human; *A. Anser*, goose; *G. Gallus*, chicken; *B. Tauru*, cow; *S. Scrofa*, pig; *F. Catus*, cat; *R. Norvegicus*, rat; and *M. Musculus*, mouse.).

The target genes of miR-33 were predicted using three online software programs, namely TargetScan, miRDB and miRCosm. The results show that 188, 164 and 422 genes were predicted. A total of 22 similar target genes were detected. With reference to the literature, we chose a target sequence area that combines with and complements the miR-33 seed sequence area. Nine target genes were selected for further validation (Figure 3): *ABCA1*, *ABCG1*, Neimann Pick C (*NPC1*), *CROT*, *HADHB*, *AMPKα1*, insulin receptor substrate 2 (*IRS2*), glutamic pyruvate transaminase (*GPT2*) and adipose differentiation-related protein (*ADRP*). The selected target genes from this region all report either one or two target miRNA-33 binding sites, as per extended analysis of the nine-target-gene sequence in the 3′ UTR region. The *CROT*, *HADHB* and *NPC1* target binding sites were more homologous in geese than in humans, mice and cattle. However, the 2–8 seed area sequence was identical in all species. These three target genes are less homologous than the six other target genes in the 3′ UTR region.

### 2.4. Amplification of the Target Sequence and Vector Construction

On the basis of the sequence of chicken genes as defined by the National Center of Biotechnology Information, we use PCR to amplify the 3′ UTR target sequences of the *ABCA1*, *ABCG1*, *NPC1*, *CROT*, *HADHB*, *CPT1A*, *AMPKα1*, *SIRT6*, *GPT2* and *ADRP* genes of Landes geese. After cutting the fragment for plastic recycling and doubling the enzyme digestion, *GPT2* and *ADRP* were digested by the restrictive enzymes Hind III and Mlu I for identification through enzyme digestion. Other genes were digested by restrictive enzymes Hind and Sac I for identification through double enzyme digestion. The results of enzyme digestion analysis and DNA sequencing indicated that the recombinant vectors were constructed successfully.

### 2.5. Verification of miR-33 Target Gene

**Figure 4:**
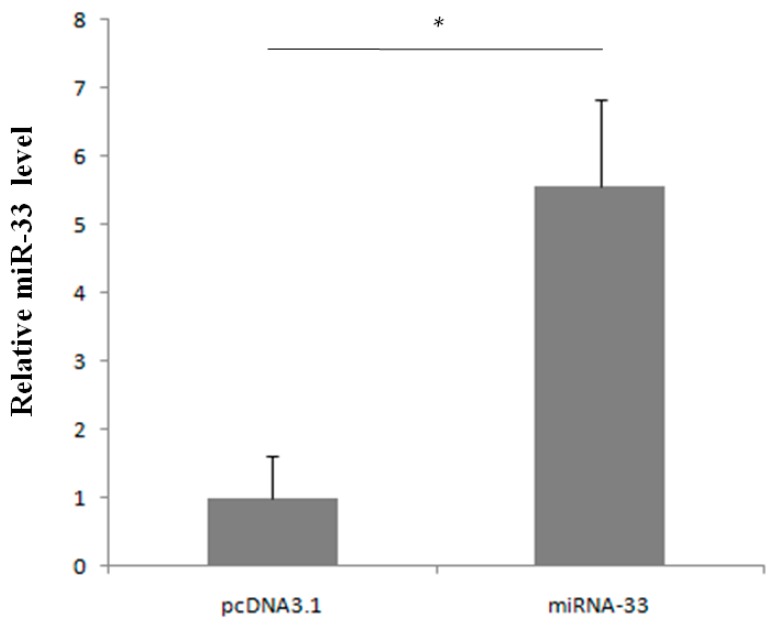
miR-33 expression in the CHO cells. * represents *p* < 0.05.

The overexpression vectors were transfected into CHO cells and used to detect miRNA-33 levels. The results showed that the expression level of miR-33 was significantly higher in the group transfected with the overexpression vector of miRNA-33 than in the control group transfected with the empty carrier pcDNA3.1 (Figure 4). Thus, miRNA-33 is successfully overexpressed and can verify the target genes.

**Figure 5:**
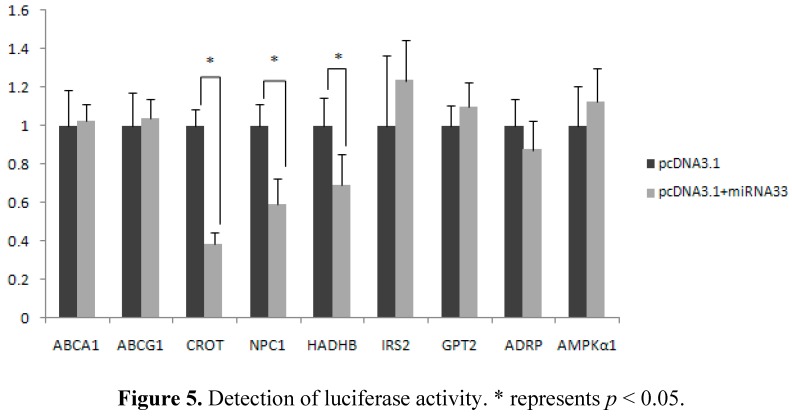
Detection of luciferase activity. * represents *p* < 0.05.

Either the overexpression vector pcDNA3.1-miRNA-33 or the control vector pcDNA3.1, the report vector pMIR-REPORT of the target genes and the internal vector PhRL-TK were cotransfected into CHO cells (Figure 5). The repeated experiment results indicate that in comparison with the control group, luciferase activity in the *ABCA1*, *ABCG1*, *IRS2*, *GPT2*, *ADRP* and *AMPKα1* genes did not change significantly in the miRNA-33 overexpression group. However, luciferase activity in the *CROT*, *NPC1* and *HADHB* genes was significantly lower in the overexpression group than in the control group (*p* < 0.05). Moreover, the inhibition rate of the *CROT* gene is highest among the genes and reaches 70%.

### 2.6. Verification of the miR-33 Target Site

**Figure 6:**
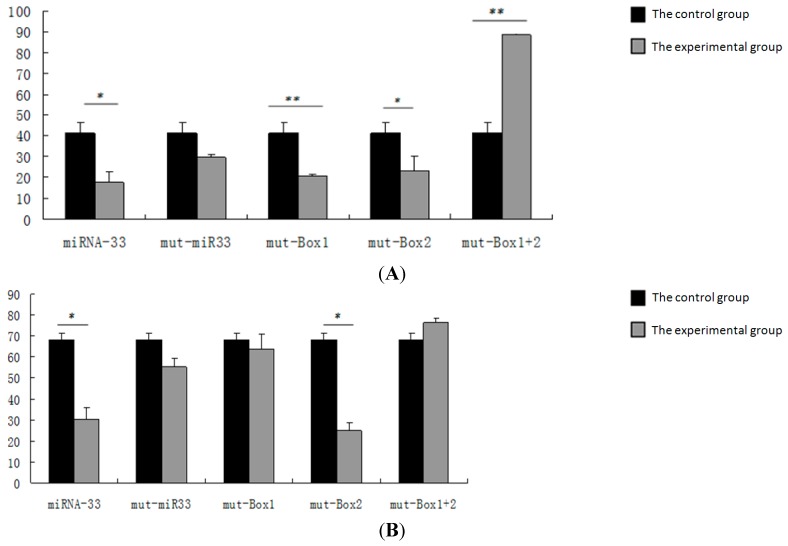

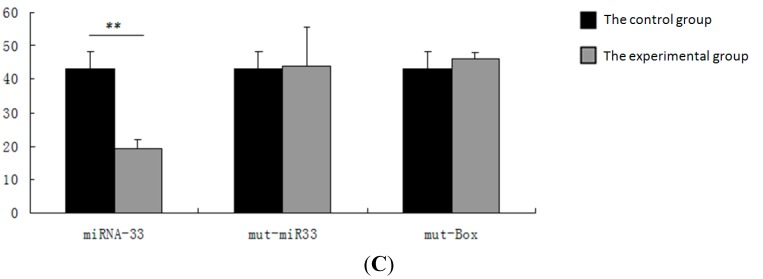
Detection of luciferase activity after target site mutation. (**A**) Luciferase activity after target site mutation in the *CROT* gene; (**B**) luciferase activity after target site mutation in the *HADHB* gene; (**C**) luciferase activity after target site mutation in the *NPC1* gene. Control group: pcDNA3.1 + pmiR-report + phRL-TK; miRNA-33: pcDNA3.1-miR-33 + pmiR-report + phRL-TK; mut-miR-33: pcDNA3.1-mut-miR-33 + pmiR-report + phRL-TK; mut-Box: pcDNA3.1-miR-33 + mut-pmir-report + phRL-TK. Mut is an abbreviation of the mutant gene. Mut-miR-33 represents the mutation of miR-33, and mut-box represents the mutation of target sites. * represents *p* < 0.05; ** represents *p* < 0.01.

The report vectors of the 2–5 base at the 5′ end of miRNA-33 and of either the 2–3 or the 5–6 base at the 3′ end of target gene are mutated. The overexpression and internal vectors are then transfected into CHO cells to detect luciferase activity (Figure 6). The repeated experiment results show that the luciferase activity in the *CROT*, *HADHB* and *NPC1* genes did not change significantly when the seed sequence points of miRNA-33 were mutated.

When target Sites 1 and 2 are mutated separately in the *CROT* gene, luciferase activity drops significantly. However, this activity was considerably higher in the overexpression group than in the control group when these target sites mutated simultaneously in this gene. When target Site 1 mutates in the *HADHB* gene, the experimental group does not differ significantly from the control group. When target Site 2 mutates in this gene, luciferase activity decreases significantly. When both target Sites 1 and 2 mutate in the *HADHB* gene, the experimental group does not vary considerably from the control group. Similarly, the experimental group does not differ significantly from the control group when the target site mutates in the *NPC1* gene.

## 3. Discussion

### 3.1. Analysis of miRNA-33 Expression in the Fatty Liver of Geese

The liver is a vital organ that plays an important role in lipid metabolism, digestion, absorption, synthesis, decomposition and transport. Fatty liver disease is a chronic liver disorder characterized by macrovesicular steatosis and can develop into hepatitis, fibrosis, cirrhosis and even hepatocellular cancer [15]. Unlike human fatty liver, goose has an excellent capacity to deposit fat in the liver, although geese do not normally develop liver fibrosis or necrosis, and the functional integrity of the hepatocytes remains intact. In agricultural production, this particular characteristic is used to produce fatty liver by short-term overfeeding (approximately 2–3 weeks) [16]. Researchers hypothesized that in the formation of goose fatty liver, the liver cells maintain a high cell proliferation capability [17]. Moreover, the liver can recover from severe hepatic steatosis to normal liver without causing any pathological damage. Therefore, goose is the optimal model animals in biomedical research for fatty liver. Additionally, the results of our study will provide important information regarding the mechanism of goose hepatic steatosis through regulation of miR-33. In this study, miR-33 expression increases significantly after 19 days of overfeeding in comparison with the control group. Previous studies have reported that miR-33 is important in cholesterol homeostasis; thus, its overexpression limits the cholesterol excretion capability of liver cells. Furthermore, the HDL cholesterol levels in mice plasma decline by 29% [18]. The overexpression of miR-33 a/b can reduce the oxidation of fatty acid in liver cells, which can, in turn, result in the accumulation of excess triglycerides in human liver cells [7,8]. miR-33 also regulates insulin signals considerably; therefore, its overexpression can weaken the expression of the *IRS2* gene. The expression of the glucose-related gene *SIRT6* is reduced simultaneously. Damage in terms of specificity formation to the *SIRT6* gene of mice liver can induce fatty liver because of the enhanced glycolysis and the synthesis of triglycerides [9,19,20]. These results suggest that miR-33 can not only regulate cholesterol metabolism, but also adjust the level of fatty acid and glucose metabolism. Hence, we believe that miR-33 may contribute significantly to the inducement of fatty liver in geese.

### 3.2. Prediction of the miRNA-33 Target Gene in Landes Geese

In this study, we derived *ABCA1*, *ABCG1*, *NPC1*, *CROT*, *HADHB*, *AMPKα1*, *IRS2*, *GPT2* and *ADRP* from the 774 target genes of miR-33 by prediction. These target sites complement one another. Moreover, the *ABCA1*, *ABCG1* and *NPC1* genes are associated with cholesterol metabolism. Studies have shown that either miRNA-33 overexpression or silence can reduce or increase the level of *ABCA1* and *ABCG1* mRNA expression in the liver. Accordingly, the HDL levels in plasma can either decrease or increase [21]. Furthermore, the *NPC1* 3′ UTR gene in humans contains two miRNA-33 binding sites. These sites can cause the protein of NPC1 to suppress liver cells and macrophages. The *CROT*, *HADHB*, *CPT1A* and *AMPKα1* genes are related to fatty acid oxidation. Previous studies have detected miRNA-33a/b binding sites in the 3′ UTR of the *CROT*, *HADHB*, *CPT1A* and *AMPKα1* genes. Moreover, the overexpression of miR-33 can limit the oxidation of fatty acid in liver cells. The inhibition of endogenous miR-33 can enhance the expression of the *CROT*, *CPT1A*, *HADHB* and *AMPK* genes, as well as enhance fatty acid oxidation [7,8]. As per the results of the current study, the *NPC1*, *CROT* and *HADHB* target points are the most conservative among all of the species. In addition, part of the sequence is important to gene function as a potential miR-33 action point.

### 3.3. Verification of the miRNA-33 Target Gene

The results of dual luciferase detection show that luciferase activity in the *CROT*, *NPC1* and *HADHB* genes was significantly lower in the experimental group than in the control group (*p* < 0.05). Furthermore, the inhibition rate of the *CROT* gene is highest and reaches 70%. The *CROT*, *HADHB* and *NPC1* genes are the target genes of miR-33 in Landes geese. Previous studies indicate that miR-33 can inhibit *CROT* and *HADHB* expression to limit the oxidation of fatty acid in the liver cells of humans and mice [22,23]. Specifically, Rayner *et al.* reported that the *NPC1* 3′ UTR gene in humans contains two miRNA-33 binding sites. The *NPC1* protein suppresses the liver cells and macrophages. Furthermore, luciferase activity in this study did not change significantly in other genes. We speculate that miR-33 does not target the inhibition of these genes, possibly because the target sequences are low in species conservatism. Thus, we infer that they are not the target genes of miR-33.

### 3.4. Verification of the miRNA-33 Target Site

To determine the role of the miRNA-33 target site, we allow the 2–5 bases at the 5′ end to mutate; therefore, they cannot combine with the target site of the target gene. Luciferase activity did not differ significantly in the *CROT*, *HADHB* and *NPC1* genes; this result suggests that miR-33 combines with the target genes according to a seed sequence to inhibit the expression of target genes. The *CROT* and *HADHB* genes display two target points on the complementary sequence of the 3′ UTR in combination with the miR-33 seed during target gene prediction. To verify the target sites of the *CROT*, *HADHB* and *NPC1* genes, we allow either the 2–3 or 5–6 base at the 3′ end of the target gene report vector to mutate. As a result, the target point cannot be combined with the miR-33 seed sequence. When target Sites 1 and 2 mutate separately in the *CROT* gene, luciferase activity decreases significantly. This finding suggests that when the sites mutate individually, another site can still be combined with the seed sequence of miRNA-33. Hence, both sites are miR-33 target sites in the *CROT* gene. When target Site 1 mutates in the *HADHB* gene, the experimental group does not vary significantly from the control group. When target Site 2 mutates in this gene, luciferase activity drops significantly. When both target Sites 1 and 2 mutate in the *HADHB* gene, the experimental group does not differ considerably from the control group. This result suggests that target Site 1 is a miR-33 target site in the *HADHB* gene, possibly because the two *HADHB* target sites are near each other. Given the easy reach of points, the miR-33 seed sequence is preferably combined with target Point 1. In addition, Gerin *et al.* obtained results for human liver cancer cells that were consistent with those of the current study [8]. The *NPC1* gene inhibits miR-33 in response to the mutations of *NPC1* target points. These findings indicate that the miR-33 seed sequences and the target sites are key to inhibiting the gene expression of miR-33. The results also suggest that the method of point mutation in this experiment is simple and effective.

## 4. Experimental Section

### 4.1. Experimental Animals and Breeding Management

Procedures involving animals and their care conformed to the U.S. National Institute of Health guidelines (NIH Pub. No. 85-23, revised 1996) and were approved by the laboratory-animal management and experimental-animal ethics committee of Yangzhou University.

Fifteen male Landes geese (Anser anser; National Waterfowl gene pool, Taizhou, China) were randomly divided into experimental (*n* = 9) and control groups (*n* = 6). All of the animals were kept in cages and fed with maize. In the experimental group, the geese were force-fed with a carbohydrate diet that consisted of boiled maize (boiled maize, 1.0% plant oil, 0.8% salt), whereas the control group was allowed free choice in feeding with maize. Three geese from each group were exsanguinated (jugular vein) after 0 (70 days old), 10 (80 days old) and 19 (89 days old) days of overfeeding. Liver samples were obtained, snap-frozen in liquid nitrogen and stored at −70 °C until needed for nucleic acid extraction.

### 4.2. Prediction of Target Genes

The online software programs TargetScan, miRDB and miRCosm were used to predict the target genes of miRNA-33 with reference to the gene sequence of chickens. The target genes predicted with the three software programs were intersected for further analysis. In accordance with the reference literature, the genes related to fatty acid oxidation and cholesterol transport were selected for target gene validation and sugar metabolism. The websites of the three software programs for target gene prediction are listed [24,25,26].

### 4.3. Primers of Carrier Construction

After predicting the target genes of miRNA-33, we chose several important target genes, such as *ABCA1*, *ABCG1*, *NPC1*, *CROT*, *HADHB*, *ADRP*, *IRS2*, *GPT2* and *AMPKα1*. According to the 3′ UTR of target genes, we designed the primers to obtain the PCR product with the target site. We further designed point mutation primers of the miR-33 target gene. Additionally, we designed a pair of amplification primers of the miR-33 precursor sequence to synthesize the miR-33 overexpression vector and two pairs of mutation primers of the miR-33 mature sequence. All primers are shown in Table 1.

**Table 1:** Primers for carrier construction.

Application	Primers (5′→3′)	Length of the Product (bp)
*ABCA1* target site	F cgagctcGCCAATTTCAGCCAAGAAGTGA	70
	R cccaagcttCTTTGGGAGTAACCTATCCCCAG	
*ABCG1* target site	F cgagctcAGGAAGAAGAAATAGAAGGGAA	267
	R cccaagcttACAGAAAACCACAAAGATGAAA	
*NPC1* target site	F cgagctcCTGGACTGCTCAACCACTGAC	211
	R cccaagcttGCCTCTCCCATTGGAATGTA	
*NPC1* target site	F AGAGACAAAAATTGCATCAACCTGCATTTA	211
	R GCAATTTTTGTCTCTATTTTTAGGGGGG	
*CROT* target site	F cgagctcATTTGCAACAGCAATGCAAG	197
	R cccaagcttAGTGCTCCACTGGCAAAAAC	
*CROT* target site 1 mutation	F ATCTCCCAAGTATGTTTGCGCTGTTGAGGCA	197
	R GCAAACATACTTGGGAGATATGGTGTTG	
*CROT* target site 2 mutation	F CCCAAGCTTAGTGCTCCACTGGCAAAAAC	197
	RCGAGCTCATTTGCAACAGCAGCGCAAGTAGTA	
*HADHB* target site	F cgagctcATGGGGGGACTGCTGAAGGAGT	256
	R cccaagcttGAGATTAGTGTGGTTACGACGA	
*HADHB* target site 1 mutation	F TGTTTTCATTAGTGCGCTGAAATGGCATTGCC	256
	R GCACTAATGAAAACATACATACAGTCCT	
*HADHB* target site 2 mutation	F TGCATTGAAATGGCGCTGCCAGGCACAGGA	256
	R TCCTGTGCCTGGCAGCGCCATTTCAATGCA	
*ADRP* target site	F cgagctcGGCTGCTGACTTGGTAGGAG	415
	R cgacgcgtCACAACCAGGCATTGCTCTA	
*IRS2* target site	F cgagctcGCCCAACTCATGTCCTGTCA	358
	R cccaagcttAGTTCAGTAAGGCTGGCGAC	
*GPT2* target site	F cgagctcACAGCAGACAGGGAACACTT	223
	R cgacgcgtATCTGCAAGTCGAAAGCCAG	
*AMPKα1* target site	F cgagctcAACAAAGGCGCTGAAAAAACTA	321
	R cccaagcttCTGAATAAAGGGGGAAGGAACA	
miR-33 overexpression	F ggaattcCCTAAAGCTGGAGCCTTCCT	203
	R ccgctcgagCGGCTCGCTATTTTAGTTGC	
miR-33 point mutation 1	F GTGCATTGTAGTTGCGCTGCATGTGACGGCA	203
	R GCAACTACAATGCACTACAGCTGCCACC	
miR-33 point mutation 2	F AGTGCATTGTAGTTGCGCAACATGTGACGG	203
	R GCGCAACTACAATGCACTACAGCTGCCA	

The underlined lowercase letters represent the *Sac*I and *Mlu*I enzyme loci; the lowercase letters without underlines denote base protection; and the underlined capital letters represent mutation bases.

### 4.4. miRNA-33 Real-Time Reverse Transcription Polymerase Chain Reaction

miRNA-33 expression level was detected with the TaqMan microRNA assay real-time fluorescent quantitative PCR technology. The fluorescence quantitative PCR reaction system consisted of the following: 7.67 μL RNase-free H_2_O, 10 μL TaqMan Universal PCR Master Mix, 1.33 μL RT product and 1 μL TaqMan small RNA assay. The reaction condition was as follows: 95 °C for 10 m, 35 cycles at 95 °C for 15 s and 60 °C for 60 s.

### 4.5. Target in Vitro Assay

#### 4.5.1. Vector Construction

The amplified PCR fragment containing target sites was connected to pMD-19T by the T vector, further inserted into the pMIR-REPORT vector by *SacI* and *MluI* digestion, and we finally obtained the pMIR-REPORT-3′ UTR recombinant plasmid. The fragment containing miR-33 precursor was cloned into expression plasmid pcDNA3.1 by *EcoRI* and *Xho I* digestion to construct the miR-33 overexpression plasmid.

#### 4.5.2. Point Mutation

According to the design primers of the seed region, we constructed the pcDNA3.1-miR-33 overexpression plasmid and pMIR-REPORT-3′ UTR recombinant plasmid containing point mutation. Different primer pairs (Table 1) were used in PCR reactions to amplify putative miR-33 target sites in the 3′ UTR of different genes and native or mutated miR-33. The PCR reaction system consists of the following: 2.5 μL 10 × Pyrobest Buffer II (Mg^2+^ Plus), 0.125 μL Pyrobest DNA Polymerase (5 U/μL), 2 μL deoxynucleotide mixture (2.5 mM), 1 μL forward primer (10 μM), 1 μL reverse primer (10 μM), 1 μL DNA and 17.375 μL ddH_2_O. The reaction conditions are as follows: 94 °C for 5 min (94 °C for 30 s, 65 °C for 30 s, 72 °C for 8 min, 16 cycles) and 72 °C for 10 min, 4 °C. *Dpn*I is applied to handle the plasmid DNA template. The reaction system consists of: 2 μL 10 × T Buffer, 1 μL Dpn I, DNA < 1 μg and a maximum ddH_2_O of 20 μL.

#### 4.5.3. Dual-Luciferase Reporter Assay

According to the instructions for the dual-luciferase reporter assay system kit (Promega, Madison, WI, USA), the luciferase-based target *in vitro* assay was applied to test whether miR-33 could bind to the 3′ untranslated region (UTR) of predicted target genes.

### 4.6. CHO Culture and Transfection

CHO cells were cultured in a CO_2_ incubator (37 °C, 5% CO_2_) with Dulbecco’s Modified Eagle’s Medium as the culture medium. The CHO cells that filled the culture bottle were digested with 0.25% trypsin for cell counting. The concentrations of pcDNA3.1, pcDNA3.1-miR-33 and the mutation plasmid of miR-33 were diluted to 100 ng/μL. Moreover, the pMIR-REPORT-3′ UTR and the mutation plasmid of 3′ UTR were diluted to 50 ng/μL, whereas PhRL-TK was diluted to 1 ng/μL. The vectors were transfected into CHO cells using the Lipofectamin 2000 transfection reagent. Dual luciferase activity was detected 48 h later.

## 5. Conclusions

(1) In this study, we first employ the information matching method to clone miR-33 in the livers of Landes geese. The expression of this gene increases significantly after 19 days of overfeeding in comparison with the control group.

(2) Using the online software programs TargetScan, miRDB and miRCosm, we predict nine miRNA-33 target genes: *ABCA1*, *ABCG1*, *NPC1*, *CROT*, *HADHB*, *AMPKα1*, *IRS2*, *GPT2* and *ADRP*.

(3) We confirm that the *CROT*, *HADHB* and *NPC1* genes are the target genes of miR-33 in geese according to the dual luciferase reporter gene system.

(4) The miR-33 seed sequence (5′ 2–8 bases) is composed of action sites that are confirmed to be the target sites of the target genes.
